# Barriers and facilitators to nicotine and cannabis vaping cessation among young adults: a qualitative study using Capability, Opportunity, Motivation, and Behavior (COM-B) model and Theoretical Domains Framework (TDF)

**DOI:** 10.1093/abm/kaaf096

**Published:** 2025-12-01

**Authors:** Nhung Nguyen, Jason M Satterfield, Salomeh Keyhani, Gregory M Marcus, Pamela M Ling

**Affiliations:** HEARTY Lab, University of California, San Francisco, San Francisco, CA, 94158, United States; Center for Tobacco Control Research and Education, University of California, San Francisco, San Francisco, CA, 94143, United States; Division of General Internal Medicine, Department of Medicine, University of California, San Francisco, San Francisco, CA, 94115, United States; Division of General Internal Medicine, Department of Medicine, University of California, San Francisco, San Francisco, CA, 94115, United States; Center for Data to Discovery and Delivery Innovation (3DI) San Francisco Veterans Affairs Health Care System, San Francisco, CA, 94121, United States; Division of Cardiology, Department of Medicine, University of California, San Francisco, San Francisco, CA, 94117, United States; Center for Tobacco Control Research and Education, University of California, San Francisco, San Francisco, CA, 94143, United States; Division of General Internal Medicine, Department of Medicine, University of California, San Francisco, San Francisco, CA, 94115, United States

**Keywords:** tobacco, marijuana, co-use, quitting, treatment, youth

## Abstract

**Background:**

Despite young adults’ growing use of nicotine and cannabis vaporized products (vaping), little is known about factors influencing vaping cessation.

**Purpose:**

Identify barriers and facilitators to nicotine and/or cannabis vaping cessation among young adults (18-29 years old).

**Methods:**

We conducted a thematic analysis of interviews with 20 California young adults (mean age = 22.8, racially and ethnically diverse) who vaped nicotine and/or cannabis (specifically delta-9-tetrahydrocannabinol or THC) in 2024-2025. We mapped cessation-related barriers and facilitators to Capability, Opportunity, Motivation, and Behavior model and Theoretical Domains Framework.

**Results:**

Young adults expressed stronger motivation to stop vaping nicotine than cannabis. *Psychological Capability* barriers involved a lack of self-control over nicotine vaping. *Physical Opportunity* factors, such as product accessibility and treatment unaffordability, hindered quitting, while the cost burden of vaporized products was a facilitator. *Social Opportunity* included both barriers (ie, socialization) and facilitators (ie, protection of loved ones or relationships). *Automatic Motivation* barriers included habitual use and addiction, while negative emotion toward vaping harms facilitated quitting. *Reflective Motivation* included the most identified factors for barriers (eg, low perceived risk of vaping, coping with mental health, and personal identity linked to vaping) and facilitators (eg, quitting desire and concerns about health and vaporized product quality). Most factors influencing vaping cessation overlapped for nicotine and cannabis. Substance-specific barriers for nicotine (ie, self-control, oral fixation, and flavor appeal) and cannabis (ie, perceived benefits) were identified.

**Conclusions:**

Findings provide insights into potential targets for future interventions to help young adults quit vaping nicotine and/or cannabis.

## Introduction

The use of vaporized nicotine and cannabis products (colloquially known as “vaping”) among US young adults (18-29 years old) is prevalent.^1–3^ In 2023, 24% of young adults reported past-month nicotine vaping and 13% reported past-month cannabis vaping.^4^ Nicotine and cannabis vaping often co-occur, with 37% of young adults who vaped nicotine and 65% of those who vaped cannabis reporting vaping both substances in the past 30 days (co-vaping) in 2022.^5^ In addition to the addiction potential of nicotine and tetrahydrocannabinol (THC), vaporized products contain toxins, heavy metals, and fine particles harmful to the lungs, heart, and brain.^6–8^ Compared to single-substance use, using both substances in the past 30 days (co-use) is linked to more harm (eg, mental health), addiction, and poorer cessation for both nicotine and cannabis.^9–11^ Developmental factors relevant to young adulthood (eg, sensation seeking, peer influence, identity exploration) may increase susceptibility to nicotine and cannabis use, making this population a priority for vaping cessation.^12^

Over half of young adults who vape nicotine are interested in quitting,^13^ and trajectories of nicotine and cannabis vaping are highly correlated.^14^ Individuals may use both substances for similar reasons (eg, socialization or stress management) or substance-specific reasons (stimulant effect from nicotine, relaxation effect from cannabis).^15^ These similarities and distinctions suggest that integrating cannabis cessation into nicotine cessation interventions may be beneficial to co-users.^9^ However, gaps in understanding how to support cessation of both substances hinder intervention development. It is unclear whether young adults want to quit or reduce use of both nicotine and cannabis, and whether factors influencing cessation differ by substance. Data on drivers of nicotine and cannabis cessation are needed to ensure that interventions meet the desires and needs of those seeking to quit.

Reviews highlighted critical gaps and suggestions in vaping cessation interventions among young adults. One review summarized barriers and facilitators to e-cigarette cessation, suggesting adaptation of cigarette smoking cessation strategies to support vaping cessation.^16^ However, barriers and facilitators to vaping cessation identified in previous studies were not linked to a conceptual framework, limiting their translation to intervention design. Another review underscored the need for a conceptual framework to understand the complexity of young adult vaping cessation and to inform potential intervention targets.^17^ This review also highlighted the need to address co-use with cannabis among those seeking to quit vaping to prevent increased use of cannabis during cessation attempts. Yet, no research to date has specifically examined factors influencing cannabis vaping cessation among young adults.

The Behavior Change Wheel framework has been increasingly used to guide the development of behavioral interventions. Its core is the Capability, Opportunity, Motivation, and Behavior (COM-B) model, which posits that the *Behavior* (eg, vaping cessation) is shaped by 3 constructs: *Capabilities* (physical and physiological, such as knowledge and skills to quit vaping), *Opportunities* (physical and social, such as access to cessation resources and supportive environments), and *Motivations* (automatic and reflective, such as perceptions about vaping and desire to quit).^18^ Each COM-B construct is further specified by the Theoretical Domains Framework (TDF), which synthesizes 33 behavioral theories and 128 constructs into 14 domains.^19^ For example, psychological capability is specified by the *Behavior Regulation* domain, social opportunity by the *Social Influences* domain, and automatic motivation by the *Habit* domain. Previous studies have applied the COM-B and TDF framework to identify drivers of behavior change and to guide the intervention design, including tobacco smoking cessation interventions.^20–22^ As such, this framework provides a systematic theoretical approach for identifying cognitive, affective, social, and environmental influences on vaping cessation. Furthermore, their constructs can be mapped onto the Behavior Change Technique Taxonomy to identify a range of potential targets to promote quitting vaping.

This study leverages qualitative data to provide a nuanced description of young adults’ desire and experience with vaping cessation of nicotine and/or cannabis. We aim to compare factors driving vaping cessation of the 2 substances by addressing the following research questions: *(1) Do young adults who vape both nicotine and cannabis want to quit vaping only 1 substance, or do they desire to quit both?* and *(2) What are the barriers and facilitators to quitting vaping each substance, comparing similarities and differences between quitting vaping nicotine and quitting vaping cannabis?* In this study, cannabis vaping refers specifically to vaping THC, not cannabidiol (CBD). We further sought to translate the study findings to inform future vaping cessation interventions by mapping the barriers and facilitators to the COM-B and TDF constructs. Findings will provide potential targets for future interventions to promote vaping cessation among young adults who vape both nicotine and cannabis.

## Methods

### Design and participants

We conducted semistructured interviews with 20 young adults in 2024-2025 as a part of a larger ecological momentary assessment (EMA) study.^11^^,^^23^ The parent study aimed to identify real-time predictors of vaping behaviors and cessation needs.^23^ Eligible participants were 18-29 years of age, resided in California, owned a smartphone, reported vaping either nicotine or cannabis at least 20 days during the past month, and intended to quit vaping either substance in the next 6 months. Participants were recruited through Instagram advertisements that linked to the study screener. Eligible participants provided electronic informed consent and were required to verify their identity by sending an ID photo or meeting via Zoom. Participants first completed an online baseline survey, and then completed EMAs via the study app for 30 consecutive days. Following smartphone-based EMA data collection, 20 participants with high EMA compliance (≥80%) were invited for in-depth interviews as a needs assessment for vaping cessation. The sample size was based on the recommendation for achieving qualitative thematic saturation.^24^ We purposely selected participants with high EMA compliance, as their greater engagement in research was expected to provide richer qualitative insights than those with lower compliance. This study was approved by the University of California, San Francisco Institutional Review Board.

### Data collection

The baseline survey collected data on demographics (eg, age, biological sex, race and ethnicity, and educational attainment), vaping-related characteristics (eg, vaping frequency, perceived harm of vaping, and attempt and intention to quit), and past 30-day use of other tobacco and cannabis products. The interviews were conducted in English by our team and audio-recorded via Zoom. The interview guide includes questions about the following domains: (1) vaping cessation desire and needs (eg, have you ever thought about quitting vaping nicotine/cannabis?), (2) reasons and context for vaping (eg, what were your most common reasons for vaping?), and (3) vaping-related perceptions (eg, what are your views on potential harms/benefits of vaping nicotine and cannabis?) (Supplementary document). During the interviews, we asked participants to clarify if they vaped THC or CBD. Most participants reported that THC was the primary substance in their vapes and generally referred to cannabis vaping when discussing THC use. The interviewers and the participants did not share similar characteristics that may have influenced participant comfort or disclosure. Each interview lasted 60 minutes. Participants received $100 via e-gift card for their participation.

### Data analysis

**Figure 1.**
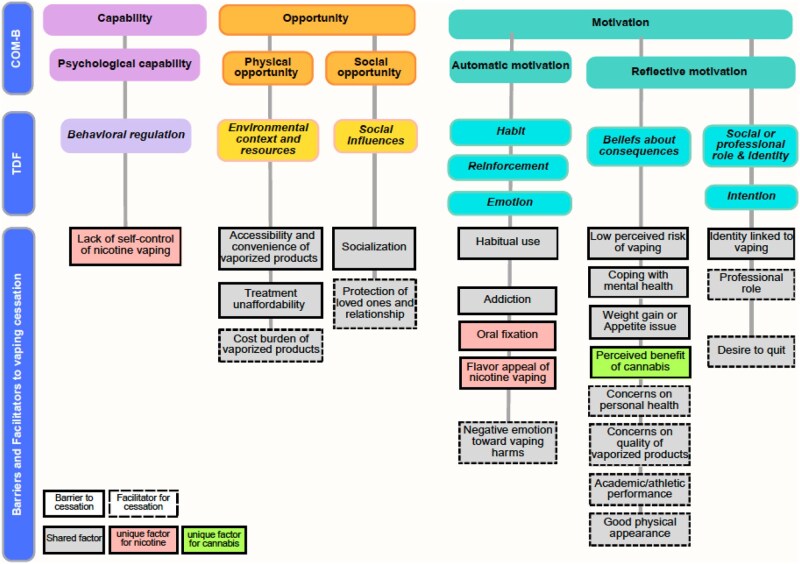
Barriers and facilitators to nicotine and cannabis vaping cessation using the COM-B and TDF model.

Audio recordings were professionally transcribed verbatim by a third-party service. Transcripts were verified for accuracy and uploaded to Dedoose for data analysis. We conducted the thematic analysis using a blended approach of deductive and inductive coding. Two researchers (N.N. and a research assistant) independently reviewed 3 transcripts to familiarize themselves with the data and developed a preliminary codebook. Deductive codes were derived from the interview guide, and inductive codes were based on insights emerging from the transcripts. After refining the codebook through discussion, we independently applied it to the remaining transcripts. We then compared coded data to reconcile discrepancies and ensure consistency. We then identified patterns across codes (eg, shared or distinct codes for nicotine versus cannabis) and organized them into themes representing barriers and facilitators to vaping cessation (eg, health concerns, habitual use, withdrawal). Thematic saturation was determined through ongoing team discussions during analysis, when repeated themes were identified and no new codes or themes emerged from subsequent transcripts. The themes were mapped to a matrix comprising the COM-B/TDF (Figure 1). We discussed and refined themes for meaningfulness, coherence, and quality. The study team had diverse backgrounds (medicine, psychology, pharmacy, and epidemiology) with extensive expertise in tobacco and cannabis use research.

## Results

### Sample baseline characteristics

The sample mean age was 22.8 years (SD = 2.5) and was racially and ethnically diverse (Table 1). Participants reported vaping nicotine on an average of 24.6 days and vaping cannabis on 17.8 days in the past 30 days. All but 1 participant reported vaping both nicotine and cannabis in the past 30 days (1 reported vaping nicotine only). Most of the participants reported vaping cannabis for recreational purposes. Participants also used other tobacco and cannabis products, with 25% smoking cigarettes and 80% smoking cannabis joints in the past 30 days. Most had tried to quit vaping nicotine (85%) or cannabis (70%) in the past 12 months, and more intended to quit vaping nicotine (85%) than cannabis (68%) within the next 6 months.

**Table 1.** Sample characteristics (*N* = 20).

Characteristics	*N* (%) or *M* (SD)
**Demographics**	
**Age (years), mean (SD)**	22.8 (2.5)
**Sex at birth**	
** Female**	11 (55.0%)
** Male**	9 (45.0%)
**Race/ethnicity**	
** NH White**	5 (25.0%)
** NH Black**	1 (5.0%)
** NH Asian**	6 (30.0%)
** Hispanic**	6 (30.0%)
** NH other/multi**	2 (10.0%)
**Education attainment**	
** Less than college**	13 (65.0%)
** College or higher**	7 (35.0%)
**Tobacco use**	
**Number of days vaping nicotine in the past 30 days, mean (SD)**	24.6 (9.8)
**Perceived harm of vaping nicotine (range 0 “Not harmful”-6 “Extremely harmful”), mean (SD)**	4.2 (1.5)
**Attempted to quit vaping nicotine in the past 12 months**	17 (85.0%)
**Intention to quit vaping nicotine**	
** Intend to quit in the next 30 days**	3 (15.0%)
** Intend to quit in the next 6 months**	14 (70.0%)
** No intent to quit in the next 6 months**	3 (15.0%)
**Past 30-day use of other tobacco products**	6 (30.0%)
** Cigarettes**	5 (25.0%)
** Cigars, cigarillos, or little cigars**	1 (5.0%)
** Smokeless tobacco**	1 (5.0%)
**Cannabis use**	
**Number of days vaping cannabis in the past 30 days, mean (SD)**	17.8 (12.2)
**Purpose for vaping cannabis**	
** Medicinal**	1 (5.0%)
** Recreational**	8 (40.0%)
** Both medicinal and recreational**	10 (50.0%)
**Perceived harm of vaping cannabis (range 0-6), mean (SD)**	2.7 (1.6)
**Attempted to quit vaping cannabis in the past 12 months**	14 (70.0%)
**Intention to quit vaping cannabis**	
** Intend to quit in the next 30 days**	3 (15.8%)
** Intend to quit in the next 6 months**	10 (52.6%)
** No intent to quit in the next 6 months**	6 (31.6%)
**Past 30-day use of other cannabis products**	17 (85.0%)
** Joint**	16 (80.0%)
** Spliff**	4 (20.0%)
** Blunt**	6 (30.0%)
** Edible**	12 (60.0%)

### Barriers and facilitators to quitting vaping nicotine and cannabis

Figure 1 illustrates how the identified barriers and facilitators to vaping cessation were mapped to the COM-B/TDF domains. While we identified themes and subthemes in all domains, most fell under the *Motivation* component: Capability (Physical: none; Psychological: 1 theme and 1 subtheme), Opportunity (Physical: 1 theme and 3 subthemes; Social: 1 theme and 2 subthemes), and Motivation (Automatic: 3 themes and 5 subthemes; Reflective: 3 themes and 11 subthemes). Many factors overlapped in quitting vaping nicotine and cannabis. Below, we outline the barriers first, followed by the facilitators within each domain, indicating whether the factors are shared between nicotine and cannabis vaping or unique to each substance. Table 2 presents exemplary quotations, comparing nicotine and cannabis vaping side by side.

**Table 2.** Barriers and facilitators to quit vaping nicotine and cannabis among young adults.

COM-B construct	TDF domain	**Barriers** ***or Facilitators***	Exemplar quotes for nicotine vaping	Exemplar quotes for cannabis vaping
**Psychological capability**	**Behavioral regulation**	**Lack of self-control of nicotine vaping** (barrier)	**V74A** [22-year-old Asian female]**:** “*Honestly, I think doing the study did kind of help me because it was like every day, I was forced to think about what I was using and how much I was using. Because, otherwise, it was like a very mindless thing. You just pick up the vape and whatever, and you’re not counting or you’re not really thinking about it.*”	Not applicable for cannabis as participants vaped limited amount for certain purposes.
**Physical opportunity**	**Environmental context and resources**	**Easy accessibility and convenience of vaporized products** (barrier)	**K18** [24-year-old Asian male]**:** “*There are a couple of vape shops in my neighborhood, so it is pretty easy to get. So even if I try to quit, I’m like a 5-minute walk from a vape store. So, it’s just easy. I mean, it’s kind of hard to stop, and it’s easy to pick back up.*”	**V36** [20-year-old White female]: “*I think it’s [cannabis vaping] just easier. You can do it in your room, in your house… It doesn’t have to bother people with that smell… And also, it’s easy to dose out. A lot of people maybe only want to hit a weed pen once and then be sort of slightly high, but they don’t have to go all the way and smoke a whole bowl, or a whole joint, or take an entire edible.*”
		**Treatment unaffordability** (barrier)	**V53** [23-year-old Hispanic male]**:** “*I used gum [to quit vaping nicotine], and I haven’t tried the patches though, I’ve tried the gum. The [nicotine] gum actually wasn’t terrible. Actually, it helped me a lot. My issue was the price of it was very high compared to everything else. So, the price was a big factor in everything*.”	**V90A** [26-year-old Black female]: “*Healthcare-wise, just to be completely honest, I don’t have health insurance, and paying out of pocket for things like that [seeking treatment], when you have so many other things to worry about, and then your kids, and making sure the house is paid for and making sure that you have gas in your car, it’s hard to find that time to take care of yourself and to kind of extend that stuff that you need for yourself. So, it ends up becoming a forethought in the background, and you’re like,* “*Okay, well, I’ll do it, but I have something else I need to do first.*”
		***Cost burden of vaporized products*** (facilitator)	**V07** [23-year-old White male]: “*It’s [nicotine vape] really just unnecessary for me. I don’t want to spend the money on it at all. And it’s not too much of a financial burden, but even just having the extra 20 bucks would be nice to just do something else with.*”	**V57** [22-year-old Asian female]: “*So cannabis is very expensive, especially when you go to an actual dispensary and buy cannabis products. There’s like three different types of taxes onto the products. So, let’s say I buy an $18 vape. It’ll total up to, like, 35 or $40, depending on the county.* “
**Social opportunity**	**Social influences**	**Socialization** (barrier)	**V60A** [19-year-old Asian female]**:** “*It gets really hard to resist it when you hang around people who have it [nicotine vape]. And especially around, where the people that I hang out with and the majority of kids of where I live, vaping and smoking has become a huge part of the socializing. And so, I feel like that’s what makes me not necessarily want to quit, just because of the socializing of it.*”	**V57** [22-year-old Asian female]: “*I feel like just because I’m in a very college environment where vaping is very prominent, weed is very prominent, it’s kind of hard to step out of my dependency on it while I’m in the environment where it is.*”
		***Protection of loved ones and relationship*** (facilitator)	**V79** [23-year-old Asian male]: “*If it is something that would change me, let’s say somebody, some of my loved ones, they say they don’t like it [vaping], then I’ll probably quit [vaping nicotine]*.”	**V98** [25-year-old White female]: “*I know that eventually they’ll get older and that’s something that I don’t want my kids to see because it’s not really brought around them even right now. So it’s not something that I want my children to think,* “*Oh, it’s—you know, it’s cool to do. I’ve seen mommy do this.*” *So yes, I do have that [thinking to quit vaping cannabis] in mind as well.*”
**Automatic motivation**	**Habits**	**Habitual use** (barrier)	**K18** [24-year-old Asian male]: “*I think it’s like the habit. I’ve been vaping every day for a while…I feel this unconscious urge to vape…I think it’s more of like a routine… I think it’s so accessible and so, like, you can do it so easily, it’s just become such like a routine habit in my life. And, yeah, that’s part of the main thing that makes it hard to quit.*”	**V92:** “*I have work at 11. If I wake up at 8:00, shower, get out at 9:00, I can smoke [vape cannabis] at 9. And I know my buzz will not last over an hour. If it won’t last past 10, I know I will not look high at work at 11. The same thing happens for when I get out of work. I don’t smoke at work. But once I’m out of work, I usually smoke [vape cannabis] with a meal. I will start cooking and I will smoke to feel high while eating.*”
	**Reinforcement**	**Addiction** (barrier)	**V35** [21-year-old Black female]: “*Almost every day. I really want to stop because I know it’s horrible for me. I have to vape at least every ten minutes. I want to make an effort [to quit vaping nicotine] but, I can’t stay away from it.*”	**V90A** [26-year-old Black female]: “*It’s [withdrawal] always with the cannabis pen, to be honest with you… The cannabis [vaping], like I mentioned, such a long time with use, that I have caught myself where I’ll be a little bit more angry. I’ll be irritable. Tired. I won’t eat. I snap a little bit, and I’m uncomfortable.*”
		**Oral fixation** (unique barrier for quitting nicotine)	**V90A** [26-year-old Black female]: “*If I have no vape or nicotine vape, I would go straight to the cannabis vape. And I’m like,* “*Okay.*” *I think it’s just I need to pick something up, put it in my mouth.*”	Not applicable for cannabis
		**Flavor appeal** (unique barrier for quitting nicotine)	**V06** [22-year-old White female]: “*I feel that’s [flavor] just kind of a bonus feature. I know that like, rationally, I can get the same sensation from eating a York Peppermint Patty, from chewing mint gum, from brushing my teeth. It’s just a very minty flavor. And I do like it. It’s something that I can do pretty much discretely and just have it instantly.*”	Participants did not use or like flavors in cannabis vapes.
	**Emotion**	***Negative emotion toward vaping harms*** (facilitator)	**V67** [20-year-old White female]: “*I think the best tactic to get me to stop would just be a video where someone scares me because of an experience that they’ve had.*”	**V63A** [24-year-old Multiracial male]: “*I think to actually emotionally impact me to a point where I maybe thought deeper about this [cannabis vaping] and wanted to make a difference [quitting], that usually happens to me more when I’m seeing a little bit more of a visceral result or a specific response…A couple of years ago, there was this big thing with THC oil vapes. It was like a bunch of fake THC oil vapes on the market. They were filled with vitamin E acetate, and it killed several teenagers. Several young adults died as a result of using fake THC oil vapes. So that could be another factor to help [quit vaping cannabis].*”
**Reflective motivation**	**Beliefs about consequences**	**Low perceived risk of vaping** (barrier)	**V10** [26-year-old White male]: “*I knew that I could do it [quitting vaping nicotine] again if I really wanted to. I just don’t have enough motivation right now. I’m not seeing any negative effects from it, and I think that’s why.*”	**V06** [22-year-old White female]: “*I would say that cannabis is positive. Like it doesn’t really cause much harm to the body…I would say, vaping cannabis is more beneficial [than nicotine], but I would say only because I truly feel the effects of it.*”
		**Coping with mental health issues** (barrier)	**V98** [25-year-old White female]: “*It’s just a sense of relief that it [nicotine vaping] gives me on a daily basis.…It’s like having, I guess you could say, a best friend right by your side. And then when they’re not around, you don’t feel comfortable. So similar to this, I feel uneasy and I don’t feel comfortable when I don’t have something that can instantly comfort me when it comes to stress or whatever it may be, even though I know that it’s not really helping, but in a sense for me it’s easing away, whatever it is at that very moment.*”	**V36** [20-year-old White female]: “*I found that in the moment doing any form of weed just quiets my brain down and numbs me from having to think about anything too hard, which is a relief when my brain is screaming at me—like all this stressful stuff, but it’s also not a solution in the slightest… It’s definitely a temporary quick fix in the moment to make myself feel better, but it’s only fixing it because it’s shutting down my brain and numbing my thoughts and not because it’s actually long-term helping my brain at all. So it’s not getting rid of the problem, it’s just like masking it for a few hours.*”
		**Weight gain or appetite issues** (barrier)	**V35** [21-year-old Black female]*:* “*I crave sugar and sweets a lot. So, once I get that sweetness [from nicotine vaping], and it lingers in my mouth for a long time, then, it just cuts my urge to want to eat… You know, I don’t gain weight from vape. This is kind of a good idea when I think about it…. I think it actually contributed to my weight loss, because I lost quite a bit of weight. It definitely curbs my appetite, I think.*”	**V57** [22-year-old Asian female]: “*I was very dependent on it for a long time before to the point where I couldn’t eat without smoking [vaping] cannabis because cannabis increases our appetite, and we eat a lot when we are high. So, I was at the point where like, oh, I need to smoke [vape] weed in order to eat.*”
		**Perceived benefit of cannabis** (unique barrier for cannabis)	Not applicable	**V98** [25-year-old White female]: “*So how everybody sees Tylenol, that’s how I see THC. If I’m in pain, or if I have a headache, or if I’m stressed out, I need to relax, I will hit the THC.*”
		***Concerns on personal health*** (facilitator)	**V74A** [22-year-old Asian female]: “*I was noticing a lot of health effects [from vaping nicotine]. I was getting headaches. I was getting shortness of breath. I’m not the healthiest person in the world to start off with. I could use a little cardio, so I was just noticing that I was getting a lot more shortness of breath quicker…. So, being forced to think about it made me think,* “*Okay, it doesn’t make me feel good.*”	**V36** [20-year-old White female]: “*I think the reason I want to quit [vaping cannabis] so bad is I just don’t like the way it makes my brain feel. I have trouble remembering things sometimes and I don’t really want to have a fried brain. I value my intelligence a lot and it’s the—I know in the long term it will really make an impact on my brain development. And so, that’s the main reason I want to stop.*”
		***Concerns on the quality of vaporized products*** (facilitator)	**V63A** [24-year-old Multiracial male]: “*The nicotine vape is much less regulated. Especially ones that I’m using. Like, these disposable ones are very unregulated. They just kind of show up in the market and we don’t—nobody really—you know, it says what the ingredients are, but there’s not really a good specific description of exactly what the chemical makeup of it is, and that is not good. That’s not something that I would consider sustainable for the long term.*”	**V74A** [22-year-old Asian female]: “*I think the weed industry just operated on an illegal level for so long that there was no regulation. And now that it’s legal, or at least in California, it’s just still not regulated enough. And I don’t know how that would work….A lot of these dispensaries, they grow their own weed and they make their own products. How do you regulate that?.*”
		***Academic or athletic performance improvement*** (facilitator)	**V07** [23-year-old White male]: “*Well, I like to do a lot of fitness activities and very much like cardiovascular sort of exercise. And my biggest factor in quitting is basically, for my lungs, my cardiovascular health is that the only thing that should be going in my lungs is oxygen air. So it’s just for the future as in my training, I guess you would say, in doing a lot of fitness things, I want to be cognizant of my lung health so vaping will not be going to make my lungs any stronger. That’s not going to make my abilities grow better. So that’s my biggest inspiration to not vape anymore.*”	**V92** [23-year-old Hispanic female]: “*As soon as I saw my grades slipping pretty drastically, I was very easily able to kind of connect the two [vaping cannabis and study] and that I would rather go and smoke [vape] outside than to spend the extra time studying or finishing an assignment … I really think it was kind of just seeing that my work ethic in school was really, really affected. And that’s just something that I would tell myself a lot because my job right now is just to be a good student and graduate*.”
		***Good physical appearance*** (facilitator)	**V35** [21-year-old Black female]: “*Only thing that’s really motivating me to want to stop vaping, like I said before, is like, my appearance so. That’s, I just don’t want to start looking, you know, just worse at such a young age or, you know, have serious health conditions. That’s about it.*”	**V57** [22-year-old Asian female]: “*I notice when I use cannabis, my acne gets worse, and I heard that cannabis kills the cells that repair our skin, and I really, really care about my skin, which is why I’ve been trying to cut down on cannabis.*”
		**Weight gain or appetite issues** (barrier)	**V35** [21-year-old Black female]: “*Once I get that sweet taste in my mouth like—I crave sugar and sweets a lot. So once I get just that sweetness, and it lingers in my mouth for a long time, then, it really, it just cuts it out, my urge to want to eat… You know, I don’t gain weight from vape, so like, this is kind of a good idea when I think about it…. I think it actually contributed to my weight loss, because I lost quite a bit of weight. It definitely curbs my appetite, I think.*”	**V57** [22-year-old Asian female]: “*So our appetites increase a lot when we feel the effects of cannabis…. So our appetites increase a lot when we feel the effects of cannabis… I was very dependent on it for a long time before to the point where I couldn’t eat without smoking cannabis because cannabis increases our appetite, and we eat a lot when we are high. So I was at the point where like, oh, I need to smoke weed in order to eat. But then after I stopped depending on it, I was able to eat regularly again.*”
	**Social or professional role and identity**	**Personal identity linked to vaping** (barrier)	**V18** [27-year-old Multiracial male]: “*I’ve sort of made vaping [nicotine] part of my personality. So, I might identify with it too much. To take that away is kind of taking away something that is so ingrained in me and something that I just do*.”	**V92** [23-year-old Hispanic female]: “*I would say that my interest in cannabis, because there’s a very big culture around cannabis, you know, you’re cooler if you smoke kind of culture.*”
		**Professional role** (facilitator)	**V67** [20-year-old White female]: “*This summer I had an internship, which was a nine to five. So obviously, I didn’t want to vape [nicotine] while I was there because it was a professional setting. So also, when I graduate, if I’m having a nine to five job and I’m in person in an office, that I think will definitely be a huge reason that I would end up stopping vaping.*”	**V92** [23-year-old Hispanic female]: “*I’d never worked with kids before… And I guess I could just kind of looked myself and says if I really am in a position of being a role model or mentor for these kids, I don’t want to have this sort of secret side or it just kind of felt wrong. So it definitely helped in kind of seeing who was the person that I kind of really wanted to be.*”
	**Intention**	***Desire to quit*** (facilitator)	**V63A** [24-year-old Multiracial male]: “*I’m 25 right now. Ideally, I’d hope that by the time I’m 30, I’m mature enough in life to move past that sort of thing because I definitely think about it actively. I actively think about stopping, and I’ve never really on the thing—like, I want to keep doing it. It’s more I just keep doing it because my brain thinks I have to or something. So I definitely plan to quit [vaping]. I just not 100% sure when that will happen.*”	**V98** [25-year-old White female]: “*I’ve actually stopped a lot of times. A lot of times when I got certain jobs in the past. But personally, because THC is not something I see as a long-term use for me. I see it as—I see it more as like as a pain relief for me. So how everybody sees Tylenol, that’s how I see THC. If I’m in pain, or if I have a headache, or if I’m stressed out, I need to relax, I will hit the THC. But it’s not something that I completely rely on and I never have*.”

#### Capability (physical or psychological capability to quit vaping)

No factors in *Physical Capability* were identified. We found a barrier, but no facilitators, in *Psychological Capability* (linked to *Behavioral Regulation* in the TDF domain). Lack of self-control of vaping was a unique barrier to quitting nicotine that was not observed with cannabis.

#### Psychological capability (TDF domain—behavioral regulation: ability to manage or change a behavior)

##### Lack of self-control for nicotine vaping (unique barrier for nicotine).

Most participants were unaware of how much nicotine they consumed, often describing vaping nicotine as a “mindless thing.” The daily self-reporting of the number of times vaping per day in the EMA collection of this study was “a wake-up call,” as **V06** [22-year-old White female] reflected, “*In my head, it’s [vaping nicotine] like, I just do this socially, I just do this when I have time. It’s not really a big thing for me. But then, being confronted with how much I was actually doing, it was like, oh, maybe this is bigger for me than I thought. Maybe I should tone it down a little.*” We did not identify this barrier in cannabis vaping, as participants were more intentional about cannabis use and vaped cannabis less frequently than they vaped nicotine. **V98** [25-year-old White female] said: “*I personally only need to have just one small hit [from cannabis vape] and I’m good for a really long time. It helps me with pain. It helps me with stress and anxiety.*”

#### Opportunity (physical and social opportunity to quit vaping)

Both barriers and facilitators were identified in this domain. Barriers to vaping cessation included both *Physical Opportunities* (TDF domain—*Environmental Context and Resources*, eg, easy accessibility/convenience of vaporized products**)** and *Social Opportunities* (TDF domain—*Social Influences*, eg, socialization). Facilitators for quitting also included *Physical Opportunities/Environmental Context and Resources* (ie, cost burden of vaporized products) and *Social Opportunities/Social Influences* (ie, protection of loved ones and relationships).

#### Physical opportunity (TDF domain—environmental context and resources: contexts or environments that enable or hinder vaping cessation)

##### Easy accessibility and convenience of vaporized products (shared barrier for nicotine and cannabis).

Participants noted that the proximity of vape shops in their neighborhoods or communities and online shopping with home delivery made purchasing vapes easy. Many also highlighted the convenience of using vaporized products anytime and anywhere, as they are discreet and unlikely to disturb others. Additionally, compared to combustible products, vaporized products were cheaper and easier to dose the desired amount, especially for cannabis.

##### Treatment unaffordability (shared barrier for nicotine and cannabis).

Participants reported being unable to afford treatment services to quit vaping, whether nicotine or cannabis, since they did not have health insurance or had to prioritize their other needs. For example, although **V53** [23-year-old Hispanic male] found nicotine gum helpful in quitting nicotine vaping, he noted that its high cost was “a big factor,” given his limited budget.

##### Cost of vaporized products (shared facilitator for nicotine and cannabis).

Participants noted that the expense of purchasing vaporized products was a financial burden since the cost added up each month, and they could save that money by stopping vaping. **V60A** [19-year-old Asian female] reflected on her cannabis vape expenses: “*I felt like, one, I was wasting my money, and two, I’m just overloading myself with the THC, and so there’s really no point*.”

#### Social opportunity (TDF domain—social influences: interpersonal processes that shape thoughts, feelings, or behaviors)

##### Socialization (shared barrier for nicotine and cannabis).

Many participants described that college settings and social circles often normalized vaping, particularly as a shared activity at parties or while hanging out with friends/roommates/colleagues. This environment made quitting challenging for young adults, as **K18** [24-year-old Asian male] said, “*I do want to quit [vaping nicotine] long term, and I’m trying to. But I think, in my social circle, people do vape, so it’s going to be kind of hard for me to just completely quit*.”

##### Protection of loved ones or relationships (shared facilitator for nicotine and cannabis).

Participants mentioned that they may quit vaping, both nicotine and cannabis, to protect their loved ones (eg, children) from vaping or to protect their relationships with partners/friends. **V02** [26-year-old Asian male] described how family could facilitate vaping cessation in general: “*I think every person’s relationship with their family is different. It’s also how your family goes about it [vaping]. Some families could be like, ‘This is bad. What are you doing?’ So that makes you not want to [vape], you know, listen. My wife is like, she wants me to live long. She doesn’t want me to be unhealthy, so I’m listening. I do think family can help [with quitting]*.”

#### Motivation (automatic or reflective motivation for vaping cessation)

Within *Automatic Motivation*, barriers included habitual use (linked to *Habit* domain of the TDF), addiction to nicotine or cannabis, oral fixation, and the flavor appeal of nicotine vaping (*Reinforcement* domain of the TDF), while a facilitator was negative emotion towards vaping harms (*Emotion* domain of the TDF). *Reflective Motivation* (*Beliefs about Consequences, Social or Professional Role and Identity, and Intention* domains of the TDF) included the most shared barriers and facilitators to vaping cessation of nicotine and cannabis. Barriers included low perceived risk of vaping, coping with mental health issues, personal identity with vaping, weight gain/appetite issues, and perceived benefit of cannabis. Facilitators included concerns about personal health, concerns about the quality of vaporized products, academic/athletic performance improvement, good physical appearance, professional role, and desire to quit.

#### Automatic motivation (TDF domains—habit, reinforcement, and emotion: habits, impulses, and emotional responses that influence motivation to quit vaping)

##### Habitual use (shared barrier for nicotine and cannabis).

Participants often vaped nicotine and cannabis as a part of their daily routines (eg, before going to work, during break, or before going to bed). **V90A** [26-year-old Black female] said, “*It’s [nicotine vaping] just a matter of habit. I’m so used to picking something up and having it near me, and taking a hit, and then putting it back, so I feel like that facilitates that urge to go back to it*.” Likewise, **V02** [26-year-old Asian male] often vaped cannabis as part of his night or weekend routines: “*It’s [cannabis vaping] for me generally at the end of the day when I want to just relax, or it’s on the weekend. You know, I just want to feel relaxed and take a chill.*”

##### Addiction to nicotine and cannabis (shared barrier for nicotine and cannabis).

Participants often expressed addiction to nicotine more than to cannabis since nicotine was “their main thing” and “way more intense” than cannabis. **V18** [27-year-old Multiracial male] described that “*vaping [nicotine] is such an addiction for me currently, to where I’m not even making a conscious decision. It’s just on me at all times.*” They experienced withdrawal symptoms when attempting to quit vaping both nicotine and cannabis, such as cravings, irritability, tiredness, mood swings, headaches, and eating/sleeping disruption. They often quit cold turkey without seeking support, and experiencing withdrawal symptoms led to relapse. **V53** [23-year-old Hispanic male] described withdrawal symptoms after a week of quitting nicotine: “*My mood wasn’t the same, and I was not feeling myself completely… I was getting cravings, big cravings. I was trying to fight them off. But eventually, it got the best of me when I had to go on vacation… So, I went on vacation and bought another vape. So, then it started again.*”

##### Oral fixation (unique barrier for nicotine).

Many participants mentioned oral fixation for nicotine vaping, as **V02** [26-year-old Asian male] described: “*I actually did some reflecting and talking to some friends and my doctor. And that’s where I came down to realize that it’s not really the nicotine that I’m addicted to at this point. It’s more of just having to hold something in my hand and using my mouth*.” **V90A** [26-year-old Black female] noted that if she quit vaping nicotine, she might switch to cannabis vaping due to oral fixation. Other mentioned that they thought of safer alternatives to fix oral fixation. For example, **V63A** [24-year-old Multiracial male] planned to buy Füm, which was advertised as a flavor air device with zero nicotine because “*The only thing that I actually considered doing to quit vaping was buying some sort of nicotine-free alternative device that still provided oral fixation*.”

##### Flavor appeal (unique barrier for nicotine).

Flavors in e-liquids reinforced the continued vaping of nicotine. Some participants mentioned that flavors, but not nicotine strength, were their “deciding factor” when picking a nicotine vape. They would miss the flavors if they quit vaping nicotine. **V74A** [22-year-old Asian female] noted that menthol/cooling flavors kept her vaping, “*They called it like ice vapes, where it cools your throat. And that’s what really kept me in it [nicotine vaping], because they tasted good. It’s like a Coca-Cola ice vape. And I really think if it wasn’t flavored, I would not have stayed with it.*” However, most participants did not use flavors in their cannabis vapes. Of a few who used flavored cannabis vapes (eg, **V98** used ice cream flavored THC vapes), they did not like them since flavors were artificial and did not enhance the high effect of cannabis, as **V63A** [24-year-old Multiracial male] said, “*That’s actually part of the reason why I wanted to only use [cannabis] flower because I didn’t really like the artificial flavoring in addition to the cannabis. But, yeah, they, all types of like fruit, the same flavors that you find in a nicotine vape, they make for cannabis vapes as well*.”

##### Negative emotional response to vaping harms (shared facilitator for nicotine and cannabis).

Participants described their emotional responses to distressing stories or news about health effects related to vaping that motivated them to quit vaping. **V36** [20-year-old White female] said, “*The things that make me more—the desire to quit the most are when I hear stories of people’s lungs collapsing and young people who have health issues and stuff like that. And seeing just like how addicted my friends get to their vapes and how dependent they are is scary.*” **V63A** [24-year-old Multiracial male] also mentioned his emotional response to the lung injury outbreak related to cannabis vapes, making him think about quitting vaping cannabis.

#### Reflective motivation (TDF domains—intention, beliefs about consequences, and social/professional role and identity: conscious intentions, beliefs, and identity that shape motivation to quit vaping)

##### Low perceived risk of vaping (shared barrier for nicotine and cannabis).

Low perceived risk of vaping nicotine and cannabis hindered cessation. This perception stemmed from either unawareness of vaping harms or a comparison of the harms between vaporized and combustible products. For example, **V67** [20-year-old White female] said: “*We see how cigarettes have caused cancer and all these things, but since we’re the generation where vaping is new, where there’s nothing for me to look at, so we need to compare it. So, I think if there was something, then it would be more motivating to quit.*” Likewise, **V53** [23-year-old Hispanic male] thought vaping cannabis was not as harmful as smoking cannabis: “*If you’re smoking cannabis flower, there is still tar and plant matter. But if you’re hitting the extract, I feel like it’s not as bad*.”

##### Coping with mental health issues (shared barrier for nicotine and cannabis).

Participants perceived vaping as a tool for coping with daily stressors, anxiety, and depression. They considered vaping as “a quick feeling of an altered state” or “instant gratification.” However, **V18** [27-year-old Multiracial male] realized a loop between nicotine vaping and anxiety: “*I’m tricking my brain into thinking that I’m controlling my stress and anxiety because you hit the nicotine, you get the dopamine release, you get the instant feeling of something else… But it is kind of ironic because when you vape so much, your heart rate gets elevated, and sometimes breathing gets difficult, which causes more anxiety. But then I find myself reacting to that anxiety by wanting to vape more…It’s almost like vaping relieves anxiety, but it causes it at the same time. So, it’s a loop*.” **V98** [25-year-old White female] noted that vaping cannabis did not really help with mental health issues: “*It’s worsening my stress and anxiety. I wake up in the morning with anxiety to the point where the first thing I have to do is run straight to the bathroom because I have nausea and I’m vomiting because of how bad my stress and anxiety are. I really hope a lot of people get to hear this. It is a scary thing that people put to the side because they think that they have this to help with the stress, but it’s not really helping at all.*”

##### Weight gain/Appetite issue (shared barrier for nicotine and cannabis).

Some participants mentioned issues related to their weight and appetites when they tried to reduce or quit vaping nicotine and cannabis. Being afraid of gaining weight made **V57** [22-year-old Asian female] relapse into vaping nicotine because she realized she was gaining a lot of weight after 2 weeks of quitting. While some participants vaped nicotine to suppress their appetite, others used cannabis to boost their appetite. Several participants mentioned that cannabis helped them to eat more. When they tried to quit cannabis, they could not eat well, which led to relapse.

##### Personal identity linked to vaping (shared barrier for nicotine and cannabis).

Participants found quitting vaping nicotine and cannabis hard because they considered vaping a part of their identity. **V23** [22-year-old Hispanic male] articulated: “*I would love a reason to quit, but I don’t know. It’s also kind of like, I feel like quitting entirely would be like leveling a piece of myself. I don’t want to make it an identity, but I’ve been at it for a long time*.” This identification made young people feel vaping cessation like a personal loss rather than a behavioral change.

##### Perceived benefits of cannabis (unique barrier for cannabis).

Participants commonly viewed cannabis as less harmful than nicotine, often citing its perceived medicinal and recreational benefits. Many reported using cannabis to self-medicate for insomnia, chronic pain, stress, and anxiety. **V53** [23-year-old Hispanic male] said: “*I believe cannabis is beneficial in a lot of ways, and I don’t really see any downsides to it, other than you can get mental cravings from it. It will also make you lazy if you smoke all day. But, I’ve never had any medical issues with it. If anything, it’s helped my medical issues. It’s helped my sleep.*” **V06** [22-year-old White female] even said that “*I would rather use cannabis as a medicine instead of taking pills*.” This perception contributed to less motivation to quit cannabis compared to nicotine.

##### Concerns about personal health (shared facilitator for nicotine and cannabis).

Participants expressed concerns about personal health, especially respiratory/cardiovascular issues related to nicotine and brain impairment related to cannabis. This concern motivated them to quit vaping to avoid adverse health effects. For example, **V53** [23-year-old Hispanic male] said: “*I’m a pretty healthy person, but I definitely believe that vapes contribute to lung issues in a way where I was coughing a lot, but I’m not to a point where it was detrimental to me…I’m honestly trying to stop nicotine completely because it has been affecting my heart rate, and I’m sure my blood pressure, you know. So, I’m trying to really cut down because it’s probably one of the hardest things I’ve done in my life, to be real. It’s a big commitment to stop.*”

##### Concerns about the quality of vaporized products (shared facilitator for nicotine and cannabis).

Participants were concerned about the unregulated quality of vaporized products, especially cannabis. They mentioned that vaporized products are “less regulated” and “there’s no transparency” on ingredients inside vapes, especially “very potent THC oil” in cannabis vapes, and thus, vaping was “overall much more of a risk.” **V74A** [22-year-old Asian female] said: “*It’s [cannabis] just legalized, so there’s not enough regulation that it’s completely safe yet. I’m in favor of it, but I think there needs to be something, some agency like an FDA of sorts, where they’re actually regulating the quality of these products because weed is so variable.*”

##### Academic/athletic performance improvement (shared facilitator for nicotine and cannabis).

Participants perceived that their performance in studying, working, and particularly playing sports would be improved if they quit vaping. **V79** [23-year-old Asian male] said, “*I used to play basketball and sports, but I already quit. So if I find my passion again to play, I would probably quit [vaping nicotine], and go back to gym and stuff*.” Likewise, **V06 [**22-year-old White female] vaped cannabis solely to relieve her period pain and avoided frequent use to prevent negative effects on her exercise: “*I don’t really want to be super high while I’m exercising and trying to be very precise in my movements.*” **V92** [23-year-old Hispanic female] decided to quit vaping cannabis after realizing that vaping was consuming her study time and causing her grades to decline. However, this facilitator was more influential in quitting nicotine than cannabis, as many participants consciously avoided cannabis when they needed to work, study, or play sports due to its impairing effects on their functionality.

##### Physical appearance (shared facilitator for nicotine and cannabis).

Interestingly, this facilitator was mentioned exclusively by female participants. They observed that nicotine and cannabis negatively affected their skin, further motivating them to reduce or quit using these substances. For example, **V74A** [22-year-old Asian female] notes “*After I vaped [cannabis], I didn’t notice a breakout, but I noticed my skin just felt a little like…you know when you eat a lot of junk food and your skin just kind of feels like oily but underneath your skin*.”

##### Professional role (shared facilitator for nicotine and cannabis).

Having a full-time job or starting work in professional settings motivated participants to quit vaping. **V57** [22-year-old Asian female] said, “*I think it’s when I get a full-time job, I feel like that’s when I’ve stepped into adulthood, where I shouldn’t rely on using any cannabis or nicotine for long-term health*.” Likewise, **V92** [23-year-old Hispanic female] tried to quit cannabis when she started a job working with students at an elementary school because cannabis use was not allowed, and she did not want to use cannabis around kids.

##### Desire to quit vaping (shared facilitator for nicotine and cannabis).

Most participants wanted to quit for good when they were older, since they said vaping nicotine or cannabis was not “*something that I would consider sustainable for the long term.*” Their desire to quit vaping nicotine was stronger than that for cannabis due to their perceptions of greater harm and addiction to nicotine. Only a few wanted to quit vaping cannabis because they were aware of their addiction to cannabis. **V36** [20-year-old White female] said: “*I really do want to quit [vaping cannabis]. It’s just very hard for me…It’s kind of ironic that I’m so sure about it, but that’s the one I’m doing so consistently. I think I don’t like the way I’m so addicted to it. I feel like it’s just impossible to stop.*” However, many participants also used other cannabis products, and some considered quitting vaping cannabis while continuing to smoke cannabis flower.

## Discussion

This qualitative study found that young adults who intended to quit vaping expressed a stronger desire to quit vaping nicotine than cannabis. We further highlighted barriers and facilitators to vaping cessation, providing key cognitive, emotional, social, and environmental factors to target in helping young adults quit vaping nicotine and/or cannabis. Our findings align with previous research on nicotine vaping cessation, highlighting common barriers (eg, product accessibility, socialization, addiction, flavor, mental health, and perceived harm) and facilitators to quitting (eg, health concerns, cost burden).^25–29^ To the best of our knowledge, no published research has specifically examined factors influencing cannabis vaping cessation among young adults. This study fills this important gap while also comparing the factors influencing quitting vaping nicotine versus cannabis, revealing both shared and distinct factors.

Most interventions on co-use of tobacco and cannabis focus on combustible products (eg, spliffs, cigarettes, joints).^9^ Among the few nicotine vaping cessation interventions,^30–33^ none address cannabis vaping.^17^ Although targeting both substances adds challenges, a review indicates that treatment for tobacco and cannabis co-use is feasible and acceptable.^9^ Our study supports integrating cannabis vaping cessation into nicotine vaping interventions as we found substantial overlap in factors influencing cessation. Existing nicotine cessation strategies could be adapted to also address cannabis, while accounting for substance-specific barriers. For nicotine vaping, interventions might emphasize self-regulation of use and provide alternatives to appealing devices and flavors. For cannabis vaping, interventions should correct the misperception of cannabis as “harmless” or “medicinal” and promote healthier, evidence-based alternatives for managing pain and sleep. Intervention delivery should align with individuals’ readiness and quit goals—whether they seek to quit vaping nicotine, cannabis, or both. Sequential treatment (supporting cessation of one substance before the other) may be effective for those who want to quit both.^9^ Leveraging digital tools (eg, smartphone apps, social media) could expand interventions’ reach, engagement, and personalization.^34^

Moreover, our participants frequently vaped nicotine and cannabis to manage mental health issues, but reported that vaping did not alleviate anxiety/depression. For some, these issues were exacerbated over time. Research has shown that using both tobacco and cannabis is linked to more frequent mental health symptoms than single substance use,^10^^,^^35^ suggesting that addressing co-vaping could have potential positive impacts on mental health. Our findings highlight the need to correct misperceptions about vaping as an effective coping mechanism for mental health and to integrate mental health support into vaping cessation programs.

This study further mapped the barriers and facilitators onto the COM-B/TDF domains, offering concrete insights for addressing co-vaping cessation among young adults. The next step of this study is to link the identified factors to 9 intervention functions (eg, education, training, environmental restructuring) that are likely to promote vaping cessation and 7 policy categories (eg, guidelines, service provision, regulation) that are likely to facilitate intervention implementation based on expert consensus.^36^ For example, people with co-vaping commonly struggled with managing cravings and withdrawals for both substances, highlighting a need for behavioral training and self-monitoring tools to strengthen *Psychological Capability/Behavioral Regulation*. Participants also described social environments that normalized vaping, pointing to the importance of training on refusal skills, restructuring the environment, and strengthening social supports to improve *Physical and Social Opportunities/Environmental context and resources* and *Social influences*. Addressing *Automatic and Reflective Motivations* through goal setting, personalized quitting plans, and education on risks of co-vaping and benefits of quitting may facilitate cessation. Moreover, policies that strengthen the regulation of vaporized products, including limits on flavors, capacity or potency, and policies that enhance access to tobacco and cannabis treatment may help promote cessation. In summary, our findings provide a clear roadmap to select theory-informed intervention components that are potentially effective to help young adults achieve co-vaping cessation.

This study has several limitations. The COM-B/TDF model primarily focuses on individual factors and does not fully capture the broader socioeconomic, cultural, and political drivers of vaping cessation. Future research may incorporate complementary frameworks that cover upstream influences to better inform the intervention development and implementation (eg, intervention mapping^37^). Our convenience sample included young adults in California—the world’s largest legal cannabis market.^38^ Patterns of nicotine and cannabis vaping in the state may differ from those in states without cannabis legalization. Moreover, our participants were motivated to quit vaping and had high EMA compliance, and thus, their perspectives may differ from those who were not motivated to quit or less engaged in research. Future research should examine factors influencing vaping cessation across a broader young adult population and geographic contexts (eg, nonmotivated users or those in prohibitionist states). Finally, our team’s research experience in tobacco and cannabis use among young adults may have shaped our attention to certain themes and data interpretation.

## Conclusions

This study found that young adults reported greater motivation to quit nicotine than cannabis. We identified shared facilitators (eg, cost savings, protecting loved ones) and barriers (eg, coping with stress/anxiety, withdrawal symptoms, socialization) for quitting vaping both substances, as well as unique factors influencing cessation of nicotine (self-control and the appealing flavors) and cannabis (self-medication for sleep/pain). The findings provide a roadmap for the development of vaping cessation interventions for young adults by mapping these barriers and facilitators to the COM-B/TDF model. Future interventions targeting co-vaping should align with individuals’ goals and address both shared and substance-specific factors influencing vaping cessation of nicotine and cannabis.

## Supplementary Material

kaaf096_Supplementary_Data

## Data Availability

Deidentified data from this study are not available in a public archive. Deidentified data from this study will be made available (as allowable according to institutional IRB standards) by emailing the corresponding author.
