# Supplementary material for: Barriers and facilitators to nicotine and cannabis vaping cessation among young adults: a qualitative study using Capability, Opportunity, Motivation, and Behavior (COM-B) model and Theoretical Domains Framework (TDF)
Source: Ann Behav Med. 2025 Dec 1;59(1):kaaf096. doi: 10.1093/abm/kaaf096 (PMC12668677; doi:10.1093/abm/kaaf096)
Supplement: kaaf096_Supplementary_Data [file kaaf096_supplementary_data.docx]

**Guide for Semi-structured Interviews**

1. **Vaping cessation desire and needs**
   1. Have you ever thought about quitting vaping nicotine? How about quitting vaping cannabis? Why do (not) you want to quit?
2. *[if want to quit both]* Do you want to quit them at the same time or which substance you want to quit first?
3. *[if want to quit one substance]* Would you switch/increase us of [the other one]?
4. *[if don’t want to quit]* Do you plan to reduce your current vaping of [nicotine, cannabis]?
5. *[if already trying to quit]* How has your experience been so far?
   1. If you were going to quit vaping soon, what would you do to successfully quit?
6. What would your motivations be for quitting vaping, if any?
7. How confident would you be in quitting [tobacco use, cannabis use, or quitting both products at the same time] using a 0 not at all confident to 7 really confident scale?
8. What would your barriers be to quitting vaping, if any?
9. What do you think would be the facilitators to support your efforts to quit?

*[Probe for similarities and differences between quitting tobacco vs. quitting cannabis]*

- 1. Have you used any other tobacco/cannabis products (e.g., combustible, edible products)?

*[If yes, then ask:]*

1. What are your main [tobacco, cannabis] products?
2. Do you use any flavored products? Which flavors in which products?
3. Where do you often buy your [tobacco, cannabis] products? *[probe for online or stores, brands, etc.]*
4. Would you switch to other [tobacco, cannabis] products if you quit vaping*? [if yes, probe for which products might be used to compensate for vaping]*
   1. Do you think there should be separate interventions for tobacco vaping cessation and cannabis vaping cessation? Why (not)? Which one should be prioritized?
5. **Reasons and context for vaping**
   1. Over the course of the study, what were your most common reasons for vaping? In which contexts did you vape most often?
   2. In the smartphone surveys you completed, common contexts/reasons of your vaping nicotine/cannabis were [list of the contexts/reasons]. Can you explain this a little more about these contexts/reasons?

*[For example, if stress was their reason for vaping: Did your stress change after vaping nicotine/cannabis? How so?]*

- 1. Can you explain more about your vaping routine?

1. How often do you vape [nicotine, cannabis] on weekdays and weekends? *[probe for times per day, puffs and duration per occasion, products used, % nicotine or THC or CBD]*
2. When and where did you find yourself vaping most often?
3. Why did you often vape at [daytime/nighttime]?
4. **Perceptions**
   1. ***Perceived addictiveness***: How addictive is [tobacco, cannabis] vaping? What makes vaping addictive (nicotine, CBD, THC, flavors, non-nicotine ingredients, non-cannabis ingredients]?

Do you believe you are personally addicted to vaping [tobacco, cannabis]?

[*If yes*] What does your own addiction look like? How much does it impact your everyday life?

[*If no*] What would an addiction to vaping [nicotine, cannabis] look like? How much would it impact someone’s everyday life if they were addicted to vaping [nicotine, cannabis]?

- 1. ***Perceived harms***: What are your views on potential harms/downsides of vaping? *[probe for impacts on mood, sleep, other health effects, and social impacts]*
  2. ***Perceived benefits:*** What are your views on the potential benefits of vaping [tobacco, cannabis]? *[probe for health effects and social impacts]*
